# The association between "hypertriglyceridemic waist" and sub-clinical atherosclerosis in a multiethnic population: a cross-sectional study

**DOI:** 10.1186/1476-511X-13-38

**Published:** 2014-02-23

**Authors:** Danijela Gasevic, Axel C Carlsson, Iris A Lesser, GB John Mancini, Scott A Lear

**Affiliations:** 1 Department of Biomedical Physiology and Kinesiology, Simon Fraser University, 2600-515 W Hastings Street, Vancouver V6B 5K3, BC, Canada; 2 Center for Family and Community Medicine, Department of Neurobiology, Care Sciences and Society, Karolinska Institutet, Alfred Nobels allé 12, Huddinge 141 83, Sweden; 3 Department of Public Health and Caring Sciences/ Section of Geriatrics Uppsala University, Uppsala, Sweden; 4 Department of Medicine, University of British Columbia, 2775 Laurel Street, Vancouver, BC V5Z 1M9, Canada; 5 Faculty of Health Sciences, Simon Fraser University, Burnaby, BC, Canada; 6 Division of Cardiology, Providence Health Care, Healthy Heart Program, 180 - 1081 Burrard Street, Vancouver, BC V6Z 1Y6, Canada

**Keywords:** Hypertriglyceridemic waist, Sub-clinical atherosclerosis, Intima-media thickness, Atherosclerotic plaque, Ethnicity

## Abstract

**Background:**

"Hypertriglyceridemic waist" (HTGW) phenotype, an inexpensive early screening tool for detection of individuals at risk for type 2 diabetes and cardiovascular disease was found to be associated with subclinical atherosclerosis in various patient populations such as those with diabetes mellitus, chronic kidney disease, and those infected with human immunodeficiency virus. However, less is known regarding an association between HTGW and subclinical atherosclerosis in the apparently healthy, multiethnic population. Therefore, the aim of the study was to explore the association between HTGW and sub-clinical atherosclerosis in an apparently healthy, multiethnic population; and to investigate whether the effect of HTGW on sub-clinical atherosclerosis persists over and above the traditional atherosclerosis risk factors.

**Methods:**

We studied 809 individuals of Aboriginal, Chinese, European and South Asian origin who were assessed for indices of sub-clinical atherosclerosis (intima-media thickness (IMT), total area and presence of carotid plaques), socio-demographic and lifestyle characteristics, anthropometrics, lipids, glucose, blood pressure, and family history of cardiovascular disease.

**Results:**

We found that, compared to individuals without HTGW and after adjusting for age, ethnicity, smoking, and physical activity; men and women with HTGW had a significantly higher: IMT (men: B (95%CI = 0.084 (0.037, 1.133), p < 0.001; women: B (95%CI) = 0.041 (0.006, 0.077), p = 0.020); and total area (men: B (95%CI = 0.202 (0.058, 0.366), p = 0.005; women: B (95%CI) = 0.115 (0.006, 0.235), p = 0.037). The association between HTGW waist and presence of plaques was significant for men (OR (95%CI) = 1.904 (1.040, 3.486), p = 0.037 vs. men without HTGW), but not for women (p = 0.284). Once analyses were adjusted for additional, traditional risk factors for atherosclerosis, the effect of HTGW on sub-clinical atherosclerosis was no longer significant.

**Conclusions:**

In conclusion, HTGW may serve as an early marker of subclinical atherosclerosis in men and women, irrespective of ethnicity. However, once individuals are assessed for all traditional risk factors for atherosclerosis, the additional assessment for HTGW is not warranted.

## Background

Atherosclerosis is a chronic progressive disease associated with high cardiovascular morbidity and mortality [1]. The diagnosis of atherosclerosis in its subclinical stage (sub-clinical atherosclerosis) is important, as it would allow for early implementation of lifestyle prevention programs that may help prevent further progression and cardiovascular events [2]. Various invasive and non-invasive arterial imaging techniques (such as intravascular sonography, B-mode ultrasonography, computed tomography and magnetic imaging) have been developed to detect and diagnose sub-clinical atherosclerosis [1]. A commonly used technique is a non-invasive B-mode ultrasonography that can determine the thickness of the intima-media layers (intima-media thickness, IMT) and characterize focal plaques. Thus, IMT carotid ultrasound is an established non-invasive method for detection of early atherosclerotic disease [3]. Longitudinal studies have shown a strong association between IMT and future risk of myocardial infarction and stroke [3,4], while in clinical trials IMT is often used to evaluate the effects of coronary heart disease risk factor interventions on atherosclerotic burden [5].

Obesity, an independent predictor of cardiovascular disease (CVD) [6,7] has been found to be associated with abnormal IMT. Namely, positive associations were reported between IMT and general obesity as measured by body mass index (BMI) [8], as well as between IMT and abdominal obesity as depicted by waist circumference (WC) and waist-to-hip ratio [8-10] or visceral adipose tissue [11]. Moreover, "hypertriglyceridemic waist" (HTGW) phenotype, an inexpensive early screening tool for detection of individuals at risk for type 2 diabetes and CVD that takes into account WC and fasting triglycerides (TG) [12-14] was found to be associated with subclinical atherosclerosis in various patient populations such as those with diabetes mellitus [15,16], chronic kidney disease [17], and those infected with human immunodeficiency virus [18]. However, less is known regarding an association between HTGW and subclinical atherosclerosis in the apparently healthy, multiethnic population. Therefore, the purpose of this study was to investigate whether HTGW is associated with measures of sub-clinical atherosclerosis (IMT, presence of plaques, and total area (a composite of IMT and plaque area)) in a multi-ethnic primary prevention cohort of men and women without pre-existing diabetes or CVD; and to explore whether this association remains after taking into account the traditional risk factors for atherosclerosis.

## Methods

### Study population

Individuals were recruited as part of the Multi-Cultural Community Health Assessment Trial (M-CHAT), a study initially designed to investigate ethnic differences in body fat accumulation and how these relate to diabetes and CVD risk [19]. Men and women of self-reported Aboriginal, Chinese, European, and South Asian origin between 30 and 65 years of age, with no weight change of more than 2 kg in three months before assessment date, and with no diagnosed CVD or on medications to treat CVD-related risk factors were eligible to participate in the study. Individuals of Chinese, European, and South Asian origin had to be living in Canada for more than three years and had to be third-generation Canadian or less in order to limit possible differences in acculturation among the immigrant groups. Individuals of Aboriginal origin were eligible for the study if they reported to have at least three grandparents of exclusive Aboriginal descent. Within each ethnic group, participants were recruited across ranges of BMI (≤24.9 (lower range), 25.0-29.9 (middle range) and ≥30 (upper range)) to examine CVD associations within a range of body sizes. However, there was a difficulty in identifying Aboriginal men with a BMI of less than 25, so Aboriginal men of any BMI were recruited. Similarly, we encountered a difficulty in the recruitment of Chinese individuals with a BMI of 30 and over; therefore, the target for the upper BMI range for Chinese study participants was changed to a BMI ≥28. Despite these modifications, the study multiethnic cohort had sufficient variation in BMI values within each ethnic group. All participants provided written informed consent for the study, and the study was approved by the Simon Fraser University Research Ethics Board.

### Participant assessment

Study participants were assessed for demographics, anthropometry, health-related behaviours, lipids, and sub-clinical atherosclerosis. Body mass index was calculated as weight in kilograms divided by height in metres squared. Waist circumference was measured at the point of maximal narrowing of the waist, and an average of two measures was calculated and recorded in centimetres. Smoking status (smoker vs. former smoker/never smoker) was self-reported. Physical activity was determined by self-report, measured using the Modifiable Physical Activity questionnaire previously used in multi-ethnic populations [20,21], and reported as the average minutes per week of leisure physical activity over the previous year. A fasting blood sample was obtained from each study participant, and TG were measured using a standard protocol by the ADVIA 1650 analyzer (Bayer Health Care, Morristown, NJ). The HTGW phenotype was defined as WC ≥ 85 cm and TG ≥ 1.5 mmol/L in females, and WC ≥ 90 cm and TG ≥ 2.0 mmol/L in males [22].

Average carotid IMT, total area, and presence of plaques were assessed as indices of sub-clinical atherosclerosis as previously described [23]. Briefly, carotid IMT was assessed by a scan of carotid arteries performed at the Prevention Clinic/Healthy Heart Program at St. Paul’s Hospital in Vancouver, Canada. Carotid arteries were examined bilaterally using a 7.5 MHz to 10 MHz linear-array transducer (Image Point HX, Hewlett Packard, Andover, MA), and individual IMT measurements were taken over a 10 mm segment in the far wall of the right and left common coronary carotid artery within 2 cm proximal to the carotid bulb. The region with the thickest IMT, excluding areas with focal lesions, was measured. All scans were thereafter transferred to the Cardiovascular Imaging Research Core Laboratory at Vancouver General Hospital to be digitized for analysis (Vascular Imager 5.0, Medical Imaging Applications, LLC, Coralville, IA). The average IMT (mm) was derived from the right and left IMT measurements. Plaques in any of the carotid segments were measured. Plaques were identified, by consensus of at least 2 observers, as wall thickness that was increased focally compared with IMT on either side of the focal area. Total area (mm^2^), a superior measure of atherosclerotic burden than IMT alone [23], was calculated as the sum of IMT area (20 mm length x average IMT for the measured length (mm)) and plaque area. Plaque area was equal to the average lesion thickness (mm) multiplied by lesion length (mm)). In case of multiple plaques, average plaque thickness was calculated by dividing the area of plaques (area of plaque 1 + area of plaque 2 + …) by the total length of plaques (length of plaque 1 + length of plaque 2 + …). The intraclass and interobserver correlation values for this method were 0.922 to 0.948 and 0.850 to 0.901, respectively [23]. For repeated measures in the same subject, the accuracy and precision were -0.001 mm (not significant compared to 0.00 mm) and 0.04 mm, respectively, for IMT and -0.21 mm^2^ (not significant compared to 0.00 mm^2^) and 3.61 mm^2^, respectively, for total area. Of importance, given that our study population consisted of apparently healthy individuals, about 50 percent of participants had no detectible plaques. Consequently, instead of plaque size, we used presence of plaques variable (any plaque present vs. plaques not present) as an outcome variable to identify whether HTGW shows any association with plaque presence in this multiethnic study population.

### Statistical analyses

Out of 822 participants initially recruited for the M-CHAT study, 13 participants were missing data for measures of sub-clinical atherosclerosis. These individuals were excluded from the study, and the final sample consisted of 809 participants. Distribution of all continuous variables was explored for normality, and non-normally distributed variables (IMT, total area, TG, physical activity, fasting blood glucose, and systolic blood pressure) were transformed using the natural logarithm. Sex differences in study characteristics were explored using an independent *t*-test for continuous variables and Chi-square test for categorical variables. The differences in study characteristics among individuals were stratified as follows: 1) Without HTGW (WC and TG not elevated); 2) Elevated TG (not elevated WC); 3) Elevated WC (not elevated TG) and 4) HTGW (elevated both TG and WC). These were analyzed using general linear models and Chi-square tests for continuous and categorical variables, respectively. Multiple linear regression analyses were used to explore the association of the stratified HTGW components with IMT and total area. Logistic regression analysis was utilized to explore the association between the stratified HTGW components and presence of plaques. Given that there was a significant interaction between the effect of sex and effect of HTGW on IMT (p = 0.013), all regression models were performed for men and women separately. However, no significant interaction between the effect of ethnicity and effect of HTGW on IMT was observed (p = 0.329), therefore, the analyses were not additionally stratified by ethnic group. All regression models were adjusted for age, ethnicity, smoking and physical activity. In order to explore whether the association between HTGW and indices of sub-clinical atherosclerosis remains after the adjustment for additional, traditional risk factors for atherosclerosis, the analyses were further adjusted for total cholesterol, high-density lipoprotein cholesterol (HDL-C), fasting blood glucose, systolic blood pressure and BMI. Regression models were explored for multicollinearity, and variance inflation indices and tolerance statistics indicate no multicollinearity in our data. All analyses were performed using Statistical Package for Social Sciences 19 (SPSS, Chicago, IL). P values ≤ 0.05 were considered significant.

## Results

Baseline characteristics of male and female participants are shown in Table 1. Significantly higher cardiovascular risk was seen among men demonstrated by larger WC, higher levels of TG, fasting blood glucose and systolic blood pressure, greater prevalence of HTGW, HDL-C, greater average carotid IMT, and larger total area. Furthermore, compared to women, men were more likely to have plaques present in carotid arteries.

**Table 1:** Baseline characteristics of the study population

**Characteristic**	**Men n = 391**	**Women n = 418**	**Significance (*p*)**
Age	46.6 ± 8.7	47.5 ± 8.8	0.130
Ethnicity			0.885
Aboriginal	90 (23.0%)	101 (24.2%)	
Chinese	101 (25.8%)	115 (27.5%)	
European	98 (25.1%)	99 (23.7%)	
South Asian	102 (26.1%)	103 (24.6%)	
Current smoker	50 (12.8%)	38 (9.1%)	0.058
Family history of CVD present (%)	171 (43.7%)	186 (44%)	0.827
Physical activity (min/week)	225 (97, 444)	208 (95, 413)	0.171
BMI	27.6 ± 4.3	27.4 ± 5.2	0.520
Waist circumference (cm)	92.7 ± 11.1	85.3 ± 12.1	< 0.001
Total cholesterol (mmol/L)	5.25 ± 0.97	5.24 ± 1.03	0.925
HDL-C (mmol/L)	1.13 ± 0.29	1.43 ± 0.36	< 0.001
Triglycerides (mmol/L)	1.47 (0.99, 2.23)	1.18 (0.83, 1.63)	< 0.001
Without HTGW	134 (35.5%)	168 (40.8%)	0.008
Elevated TG	31 (8.2%)	57 (13.8%)	
Elevated WC	128 (34.0%)	117 (28.4%)	
HTGW	84 (22.3%)	70 (17%)	
Glucose (mmol/L)	5.30 (5.00, 5.60)	5.10 (4.80, 5.40)	< 0.001
Systolic blood pressure (mm Hg)	117 (110, 124)	115 (106, 125)	0.035
Intima-media thickness (mm)	0.68 (0.60, 0.77)	0.63 (0.58, 0.70)	< 0.001
Total area (mm^2^)	16.60 (13.14, 23.99)	14.24 (12.00, 20.51)	< 0.001
Presence of plaque	213 (54.5%)	191 (45.7%)	0.008

Categorical variables presented as n (%). Normally distributed continuous variables presented as mean ± SD. Skewed continuous variables presented as median (25%, 75%). Sex differences in continuous and categorical variables explored by independent *t*-test and Chi-square test, respectively. HDL-C: high-density lipoprotein cholesterol; CVD: cardiovascular disease.

Characteristics of participating men, stratified by the presence of HTGW components, are presented in Table 2. In men, HTGW phenotype was most prevalent among South Asians (32.1%) and least prevalent among Chinese (17.9%). Furthermore, the greatest percentage of men without HTGW was highest among Chinese (37.3%) which was more than three times higher than that of Aboriginals. However, prevalence of Elevated TG phenotype was about 20 times higher in Chinese men compared to their Aboriginal and European counterparts. Highest BMI, fasting blood glucose and systolic blood pressure, and lowest HDL-C were observed among men with HTGW. In addition, IMT and total area were significantly higher in men with HTGW and Elevated WC phenotypes compared to their counterparts without HTGW and those with Elevated TG.

**Table 2:** Characteristics of participating men stratified by presence of HTGW components**

**Characteristic**	**Without HTGW n = 134**	**Elevated TG n = 31**	**Elevated WC n = 128**	**HTGW n = 84**	**Overall significance (*p*)**
Age (years)	46.7 ± 8.9	46.1 ± 7.9	47.9 ± 8.7	45.5 ± 8.8	0.256
Ethnicity					< 0.001
Aboriginal	15 (11.2%)	1 (3.2%)	38 (29.7%)	25 (29.8%)	
Chinese	50 (37.3%)	19 (61.3%)	16 (12.5%)	15 (17.9%)	
European	40 (29.9%)	1 (3.2%)	39 (30.5%)	17 (20.2%)	
South Asian	29 (21.6%)	10 (32.3%)	35 (27.3%)	27 (32.1%)	
Current smoker	10 (7.5%)	3 (9.7%)	14 (10.9%)	15 (17.9%)	0.125
Family history of CVD present (%)	59 (44.0%)	14 (45.2%)	58 (45.3%)	36 (42.9%)	0.987
Physical activity (min/week)*†	238 (194, 290)	153 (99, 236)	220 (179, 272)	183 (142, 236)	0.249
Body mass index*	24.2 (23.6, 24.7)	24.7 (23.6, 25.8)	29.8 (29.2, 30.3)	30.7 (30.1, 31.4)	< 0.001^b, c, d, e, f^
Total cholesterol (mmol/L)*	5.11 ± 0.95	5.81 ± 1.01	5.00 ± 0.87	5.66 ± 0.95	< 0.001^a, c, d, f^
HDL-C (mmol/L)*	1.28 ± 0.30	0.97 ± 0.19	1.13 ± 0.27	0.94 ± 0.20	< 0.001^a, b, c, d, f^
Glucose (mmol/L)*	5.10 (4.90, 5.43)	5.20 (5.00, 5.40)	5.30 (5.10, 5.70)	5.60 (5.20, 5.90)	< 0.001^b, c, e, f^
Systolic blood pressure (mm Hg)*	115 (108, 120)	116 (108, 124)	118 (112, 126)	120 (114, 131)	0.014^c^
Intima-media thickness (mm)*†	0.67 (0.65, 0.69)	0.65 (0.61, 0.69)	0.71 (0.69, 0.73)	0.72 (0.70, 0.75)	< 0.001^b, c, d, e^
Total area (mm^2^)*†	17.87 (16.53, 19.34)	18.56 (15.75, 21.85)	20.41 (18.84, 21.85)	21.05 (19.07, 23.27)	< 0.001^b, c^
Presence of plaque	66 (49.3%)	17 (54.8%)	74 (57.8%)	53 (63.1%)	0.225

*Age adjusted, presented as mean (95%CI), † geometric means; age presented as mean ± SD; Differences in continuous variables across categories of HTGW components variable explored by general linear modelling. Categorical variables presented as n (%). Associations between categorical variables and HTGW components variable explored by Chi-square test.

Pairwise comparisons: ^a^p < 0.05, Normal WC/Normal TG vs. Elevated TG/Normal WC; ^b^p < 0.05, Normal WC/Normal TG vs. Elevated WC/Normal TG; ^c^p < 0.05, Normal WC/Normal TG vs. HTGW; ^d^p < 0.05, Elevated TG/Normal WC vs. Elevated WC/Normal TG; ^e^p < 0.05, Elevated TG/Normal WC vs. HTGW; ^f^p < 0.05, Elevated WC/Normal TG vs. HTGW.

**Elevated waist circumference (WC) was ≥ 90 cm. Elevated triglyceride (TG) levels were ≥ 2 mmol/L. Fourteen male participants are missing data for one or both hypertriglyceridemic waist (HTGW) components.

Characteristics of participating women, stratified by the presence of HTGW components, are presented in Table 3. Ethnic differences in the distribution of HTGW components in women were similar to those in men with the highest prevalence of Without HTGW and lowest prevalence of HTGW phenotypes being among Chinese women. Women with HTGW were about 5 times more likely to smoke compared to women without HTGW. In addition, BMI, fasting blood glucose, systolic blood pressure, IMT and total area were significantly higher, while HDL-C was significantly lower, in women with HTGW than in those without HTGW.

**Table 3:** Characteristics of participating women stratified by presence of HTGW components**

**Characteristic**	**Without HTGW n = 168**	**Elevated TG n = 57**	**Elevated WC n = 117**	**HTGW n = 70**	**Overall significance (*p*)**
Age (years)	48.1 ± 8.8	47.6 ± 9.7	47.3 ± 8.6	46.9 ± 8.8	0.795
Ethnicity					< 0.001
Aboriginal	19 (11.3%)	12 (21.1%)	46 (39.3%)	21 (30.0%)	
Chinese	61 (36.3%)	21 (36.8%)	24 (20.5%)	9 (12.9%)	
European	43 (25.6%)	6 (10.5%)	27 (23.1%)	21 (30.0%)	
South Asian	45 (26.8%)	18 (31.6%)	20 (17.1%)	19 (27.1%)	
Current smoker	7 (4.2%)	4 (7.0%)	10 (8.5%)	14 (20.0%)	0.001
Family history of CVD present (%)	79 (47.0%)	25 (43.9%)	48 (41.0%)	31 (44.3%)	0.798
Physical activity (min/week)*†	214 (183, 252)	197 (148, 260)	178 (147, 216)	162 (127, 208)	0.124
Body mass index*	23.8 (23.2, 24.3)	25.3 (24.3, 26.3)	31.0 (30.3, 31.7)	31.8 (30.9, 32.7)	< 0.001^a, b, c, d, e^
Total cholesterol (mmol/L)*	5.11 ± 1.03	5.71 ± 1.13	4.99 ± 0.93	5.62 ± 0.87	< 0.001^a, c, d, f^
HDL-C (mmol/L)*	1.58 ± 0.36	1.32 ± 0.36	1.40 ± 0.30	1.23 ± 0.27	< 0.001^a, b, c, f^
Glucose (mmol/L)*	4.90 (4.70, 5.30)	5.10 (4.80, 5.40)	5.20 (4.90, 5.50)	5.30 (4.90, 5.60)	< 0.001^b, c^
Systolic blood pressure (mm Hg)*	111 (104, 122)	114 (107, 125)	119 (109, 131)	120 (113, 127)	< 0.001^b, c^
Intima-media thickness (mm)*†	0.63 (0.62, 0.64)	0.66 (0.64, 0.69)	0.65 (0.64, 0.67)	0.65 (0.64, 0.67)	< 0.001^a, b, c^
Total area (mm^2^)*†	15.60 (14.76, 16.46)	16.76 (15.27, 18.41)	16.53 (15.49, 17.65)	17.46 (16.05, 18.99)	< 0.001^c^
Presence of plaque	75 (44.6%)	24 (42.1%)	52 (44.4%)	37 (52.9%)	0.595

*Age adjusted, presented as mean (95%CI), †geometric means; age presented as mean ± SD; Differences in continuous variables across categories of HTGW components variable explored by general linear modelling. Categorical variables presented as n (%). Associations between categorical variables and HTGW components variable explored by Chi-square test.

Pairwise comparisons: ^a^p < 0.05, Normal WC/Normal TG vs. Elevated TG/Normal WC; ^b^p < 0.05, Normal WC/Normal TG vs. Elevated WC/Normal TG; ^c^p < 0.05, Normal WC/Normal TG vs. HTGW; ^d^p < 0.05, Elevated TG/Normal WC vs. Elevated WC/Normal TG; ^e^p < 0.05, Elevated TG/Normal WC vs. HTGW; ^f^p < 0.05, Elevated WC/Normal TG vs. HTGW.

**Elevated waist circumference (WC) was ≥ 85 cm. Elevated triglyceride (TG) levels were ≥ 1.5 mmol/L. Six female participants are missing data for one or both hypertriglyceridemic waist (HTGW) components.

**Figure 1:**
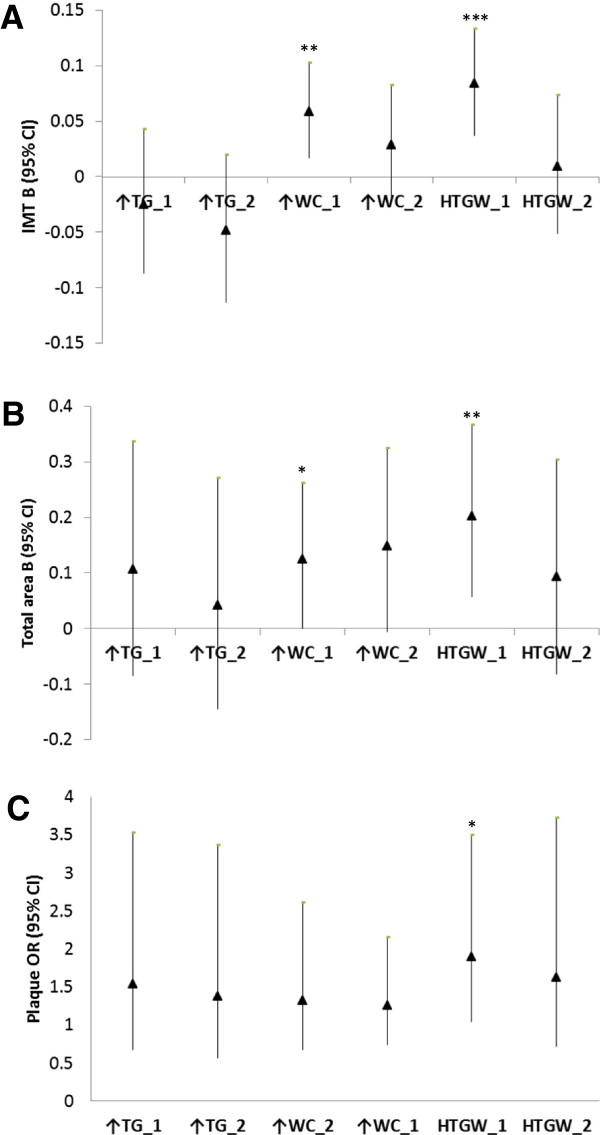
**The association between HTGW phenotype and indices of subclinical atherosclerosis in men.** Panel **A**: The association HTGW phenotype and IMT; Panel **B**: The association between HTGW phenotype and total area; Panel **C**: The association between HTGW phenotype and presence of plaques; ↑TG: Elevated triglycerides, not elevated waist circumference; ↑WC: Elevated waist circumference, not elevated triglycerides; HTGW: elevated both triglycerides and waist circumference; _1: Model 1, adjusted for age, ethnicity, smoking and physical activity; _2: Model 2 = Model 1 + additional adjustment for systolic blood pressure, total cholesterol, HDL-C, fasting blood glucose, family history of CVD, and BMI; B (95%CI): Beta unstandardized regression coefficient with a corresponding 95 percent confidence interval; OR (95%CI): odds ratio with a corresponding 95 percent confidence interval; *p < 0.05, **p < 0.01, ***p < 0.001.

The association of HTGW with indices of sub-clinical atherosclerosis among men after adjusting for age, ethnicity, smoking, and physical activity is presented in Figure 1. Intima-media thickness was significantly higher in men with Elevated WC (B (95%CI = 0.059 (0.017, 0.102), p = 0.005) and with HTGW (B (95%CI = 0.084 (0.037, 1.133), p < 0.001) compared to their counterparts without HTGW. Similarly, larger total area (a composite of IMT and plaque area) was observed among men with Elevated WC ((B (95%CI = 0.124 (0.001, 0.261), p = 0.047) and HTGW ((B (95%CI = 0.202 (0.058, 0.366), p = 0.005) then among men without HTGW. No statistically significant difference in IMT and total area was observed between men with Elevated TG and those without HTGW. Moreover, while no difference in prevalence of carotid artery plaques was noted between men without HTGW and those with Elevated TG and Elevated WC; prevalence of carotid artery plaques was significantly higher among men with HTGW than among their counterparts without HTGW (OR (95%CI) = 1.904 (1.040, 3.486), p = 0.037). However, after additional adjustment for total cholesterol, HDL-C, glucose, systolic blood pressure, BMI, and family history of CVD, traditional risk factors for atherosclerosis, the association of HTGW with indices of atherosclerosis was no longer significant (Additional file 1).

**Figure 2:**
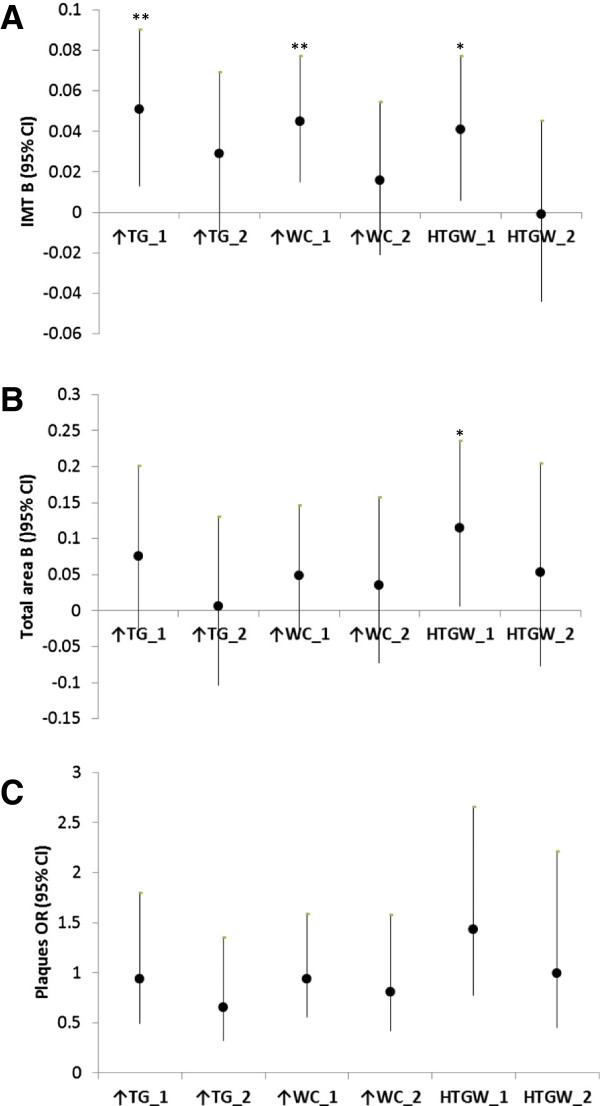
**The association between HTGW phenotype and indices of subclinical atherosclerosis in women.** Panel **A**: The association HTGW phenotype and IMT; Panel **B**: The association between HTGW phenotype and total area; Panel **C**: The association between HTGW phenotype and presence of plaques; ↑TG: Elevated triglycerides, not elevated waist circumference; ↑WC: Elevated waist circumference, not elevated triglycerides; HTGW: elevated both triglycerides and waist circumference; _1: Model 1, adjusted for age, ethnicity, smoking and physical activity; _2: Model 2 = Model 1 + additional adjustment for systolic blood pressure, total cholesterol, HDL-C, fasting blood glucose, family history of CVD, and BMI; B (95%CI): Beta unstandardized regression coefficient with a corresponding 95 percent confidence interval; OR (95%CI): odds ratio with a corresponding 95 percent confidence interval; *p < 0.05, **p < 0.01, ***p < 0.001.

Figure 2 features the association of HTGW with indices of sub-clinical atherosclerosis in women. After adjusting for age, ethnicity, smoking, and physical activity, IMT was significantly higher in women with Elevated TG (B (95%CI) = 0.051 (0.013, 0.090), p = 0.007), Elevated WC (B (95%CI) = 0.045 (0.015, 0.077), p = 0.003), and HTGW (B (95%CI) = 0.041 (0.006, 0.077), p = 0.020) than in women Without HTGW. Furthermore, total area of women with HTGW was significantly larger than that among women Without HTGW (B (95%CI) = 0.115 (0.006, 0.235), p = 0.037). However, no significant difference in total area was found between women with Elevated TG and Elevated WC and women Without HTGW. Additionally, presence of carotid artery plaques among women with Elevated TG, Elevated WC and HTGW did not significantly differ from their counterparts Without HTGW. Once analyses were additionally adjusted for traditional risk factors for atherosclerosis, HTGW phenotype was no longer associated with IMT and total area (Figure 2, Additional file 2).

## Discussion

In an apparently healthy multi-ethnic population of Aboriginal, Chinese, European and South Asian men and women, we explored the association between HTGW and indices of subclinical atherosclerosis. The findings of our study indicate that HTGW was significantly and positively associated with IMT and total area in both men and women. The positive association between HTGW and presence of plaques was significant for men; but it did not reach statistical significance for women. Significant associations between HTGW and indices of subclinical atherosclerosis were independent of age, ethnicity, smoking behaviour, and levels of physical activity. However, once analyses were adjusted for all traditionally measured risk factors for atherosclerosis, the associations of HTGW with indices of subclinical atherosclerosis were no longer significant.

Our study is congruent with studies performed in populations with diabetes mellitus [15,16], chronic kidney disease [17], and those infected with human immunodeficiency virus [18] that found a positive association between HTGW and subclinical atherosclerosis. In our study, as indicated by total area and presence of plaques, individuals with either Elevated WC or Elevated TG had more carotid atherosclerosis than individuals without HTGW; while greatest carotid atherosclerosis was observed among men and women with HTGW suggesting that the combination of a large WC and elevated TG results in greater CVD risk. Indeed, the phenotype of HTGW has previously been shown to be associated with elevated cardiometabolic risk factors [13,24-28]. Additionally, multiple prospective studies reported an increase in risk of developing cardiovascular disease [22,29-31] and diabetes [32,33] among individuals with HTGW phenotype further highlighting the utility of HTGW as a preventative screening tool.

It has been proposed that elevated fasting TG, in the presence of increased WC, represent a marker of individual’s relative inability to store energy surplus in the subcutaneous adipose tissue (a protective metabolic sink); consequently, HTGW could be a simple marker of visceral adipose tissue (dysfunctional adipose tissue) and its metabolic complications resulting from insulin resistance [14,34]. Indeed, it has recently been reported that, among subjects with type 2 diabetes, HTGW identifies subset of individuals with greater degree of visceral adiposity and subclinical atherosclerosis (15). Of importance, although there is an established link between insulin resistance and coronary artery disease (CAD) [35], presence of HTGW was found to be associated with increased CAD risk in both normoglycemic and insulin resistant individuals [36].

The rationale for measuring and interpreting WC along with TG when screening for individuals at increased cardiometabolic risk is due to a fact that not all individuals with increased WC have elevated VAT and are at increased risk for CAD (14). Indeed, it has been shown that isolated increase in WC or isolated hypertriglycerideamia showed no association with increase in CAD risk, while simultaneous presence of elevated WC and fasting TG (HTGW) was associated with a significant increase in CAD disease (13). Additionally, although cardiovascular risk of men was significantly higher than that of women in our study, which is most likely due to differences in body fat accumulation between sexes [37,38], greatest atherosclerosis was observed in both men and women with HTGW compared to their counterparts with isolated elevated WC or TG. Thus, simultaneous measurement and interpretation of WC and fasting TG can be a powerful tool to identify both men and women characterized by the atherogenic triade (hyperinsulinemia, elevated apolipoprotein B, and small, dense LDL-C) and at high risk for CAD (13, 27).

The strength of our study lies in exploring the association between HTGW and subclinical atherosclerosis in an apparently healthy and a multiethnic population, in using several markers of subclinical atherosclerosis (IMT, total area and presence of carotid plaques), and exploring whether the effect of HTGW on subclinical atherosclerosis persists over and above traditional risk factors for atherosclerosis. Our findings that HTGW phenotype is associated with subclinical atherosclerosis in apparently healthy men and women, irrespective of ethnicity, suggests an important role that HTGW may play in primary care practice in identifying patients at risk for atherosclerosis. The use of HTGW would largely expedite and simplify triaging of patients by identifying those "in need" of aggressive atherosclerosis prevention, thereby decreasing the amount of time and complex testing a primary health care practitioner would spend screening for such patients and potentially translate in lower health care costs. Moreover, this quick assessment would further allow a primary care health practitioner to spend more time in counselling a patient on how to decrease their risk for atherosclerosis.

Patients seen by specialists often already have documentation of traditional risk factors and so further assessment for HTGW may not necessarily add information toward atherosclerosis risk since we observed the effect of HTGW on subclinical atherosclerosis was no longer significant once all traditional atherosclerosis risk factors were added to the model. However, given a strong association of HTGW with cardiometabolic risk factors [12,13,24-28] and its ability to predict cardiovascular disease event [30], HTGW may be used as a simple and inexpensive tool in clinical practice to monitor patients’ changes (improvement/deterioration) in cardiometabolic risk. Additionally, this simple tool could also provide greater incentive to patients for lifestyle change, as they can easily track the effect of lifestyle changes on their cardiometabolic risk.

Despite its potential benefits in identifying individuals at increased risk for atherosclerosis, the widespread use of HTGW as a screening tool may be limited due to the lack of routine measurement of WC in clinical practice. Indeed, in a recently published study, Gupta et al. (2012) surveyed Canadian primary care physicians and found that WC was routinely measured by only 6% of physicians [39]. This finding contrasts with the knowledge that 80% of surveyed physicians considered WC to be a vital sign [39]. This discrepancy may be due to a notion amongst clinicians that WC is a tool for research purposes only; a lack of effort to continuously inform physicians about the clinical usefulness of the routine WC measurements; and a lack of a standardized protocol for WC measurement [40]. There is a concern that a lack of standardized protocol for measurement of WC may influence clinical decision-making [41-43]. However, it has been reported that the reproducibility of WC measurement is high regardless of the measurement site [44] and that the WC measurement protocol has no significant influence on the association of WC with CVD, diabetes, all-cause and CVD mortality. Given the simplicity, high reproducibility of and low costs associated with the WC measurement, it has been argued that WC should be used by the medical community more consistently [28].

Ethnic-specific WC targets may pose an additional challenge in identifying individuals with HTGW and hence at risk for atherosclerosis [17]. While using current definition for HTGW [22], we found that ethnicity did not modify the association between HTGW and indices of atherosclerosis; however, our results indicate significant ethnic differences in the distribution of HTGW components which is most likely driven by body fat accumulation shown to be different in distinct ethnic groups [45,46]. Thus, we believe that further exploration on the role of ethnicity in the association between HTGW and subclinical atherosclerosis is warranted given the already established ethnic differences in CVD risk [47,48].

There are limitations to the study design as it is cross-sectional rather than prospective. Future research should employ a prospective study design to determine causal relationships. Our study sample may not be representative of a general population given that our participants were between ages of 30 and 65 and purposely recruited across a range of BMI values. However, this approach to recruitment may be advantageous, as it allowed us to explore the association between HTGW and atherosclerosis in a population with diverse body sizes. Furthermore, due to a difficulty in identifying individuals of Chinese origin with BMI of 30 and higher, the target for the upper BMI range of Chinese individuals was changed to a BMI of 28 or higher. However, we believe that this slight modification in the recruitment strategy did not influence our study results where Chinese individuals were shown to have lower prevalence of HTGW and hence lower cardiovascular risk compared to their ethnic counterparts; as already reported in the literature [48,49].

## Conclusions

In conclusion, in a multi-ethnic population of apparently healthy individuals, in both men and women, the presence of HTGW was positively associated with subclinical atherosclerosis as depicted by carotid IMT and total area indices after adjustment for age, ethnicity, smoking and physical activity. However, in our study, HTGW did not show the association with subclinical atherosclerosis over and above the traditional risk factors for atherosclerosis. Our results further verify the importance of HTGW as a simple clinical tool that may serve as an early marker of subclinical atherosclerosis in men and women, irrespective of ethnicity. The implementation of HTGW as a preventative clinical tool would allow for timely implementation of prevention programs to reduce subclinical atherosclerosis and future CVD risk. However, once individuals are assessed for all traditional risk factors for atherosclerosis, the results of our study indicate that the additional assessment of men and women for HTGW is not warranted.

## Abbreviations

BMI: Body mass index; CAD: Coronary artery disease; CVD: Cardiovascular disease; HDL-C: High density lipoprotein cholesterol; HTGW: Hypertriglyceridemic waist; IMT: Intima-media thickness; M-CHAT: Multi-Cultural Community Health Assessment Trial; TG: Triglycerides; WC: Waist circumference.

## Competing interests

The authors declare that they have no competing interests.

## Authors’ contributions

DG performed statistical analyses, data interpretation and drafted the manuscript. ACC helped with the interpretation of data and drafting the manuscript. IAL participated in the discussion and critically revised the manuscript. GBJM and SAL have made substantial contributions to conception and design of the study, data interpretation and critically revised the manuscript. All authors read and approved the final manuscript.

## Supplementary Material

Additional file 1 The association of HTGW with IMT, total area and presence of carotid artery plaques in men. Click here for file

Additional file 2 The association of HTGW with IMT, total area and presence of carotid artery plaques in women. Click here for file

## References

[B1] Toth PP Subclinical atherosclerosis: what it is, what it means and what we can do about it Int J Clin Prac 2008 62 1246 1254 10.1111/j.1742-1241.2008.01804.x PMC2658007 18564201

[B2] Miller M An emerging paradigm in atherosclerosis: focus on subclinical disease Postgrad Med 2009 121 49 59 10.3810/pgm.2009.03.1976 19332962

[B3] Bots ML Hoes AW Koudstaal PJ Hofman A Grobbee DE Common carotid intima-media thickness and risk of stroke and myocardial infarction: the Rotterdam Study Circulation 1997 96 1432 1437 10.1161/01.CIR.96.5.1432 9315528

[B4] O’Leary DH Polak JF Kronmal RA Manolio TA Burke GL Wolfson SK Jr Carotid-artery intima and media thickness as a risk factor for myocardial infarction and stroke in older adults Cardiovasc Health Study Collaborative Res Group NEJM 1999 340 14 22 10.1056/NEJM199901073400103 9878640

[B5] Bots ML Grobbee DE Intima media thickness as a surrogate marker for generalised atherosclerosis Cardiovasc Drugs Ther 2002 16 341 351 10.1023/A:1021738111273 12652104

[B6] Hubert HB Feinleib M McNamara PM Castellj WP Obesity as an independent risk factor for cardiovascular disease: a 26-year follow-up of participants in the Framingham Heart Study Circulation 1983 67 968 977 10.1161/01.CIR.67.5.968 6219830

[B7] Rabkin SW Mathewson FA Hsu PH Relation of body weight to development of ischemic heart disease in a cohort of young North American men after a 26 year observation period: the Manitoba Study Am J Cardiol 1977 39 452 458 10.1016/S0002-9149(77)80104-5 842466

[B8] De Michele M Panico S Iannuzzi A Celentano E Ciardullo AV Galasso R Saccheti L Zarrilli F Bond MG Rubba P Association of obesity and central fat distribution with carotid artery wall thickening in middle-aged women Stroke 2002 33 2923 2928 10.1161/01.STR.0000038989.90931.BE 12468792

[B9] Hassinen M Lakka TA Komulainen P Haapala I Nissinen A Rauramaa R Association of waist and hip circumference with 12-year progression of carotid intima-media thickness in elderly women Int J Obes (Lond) 2007 31 1406 1411 10.1038/sj.ijo.0803613 17372615

[B10] Lakka TA Lakka HM Salonen R Kaplan GA Salonen JT Abdominal obesity is associated with accelerated progression of carotid atherosclerosis in men Atherosclerosis 2001 154 497 504 10.1016/S0021-9150(00)00514-1 11166785

[B11] Lear SA Humphries KH Kohli S Frohlich JJ Birmingham CL Mancini GB Visceral adipose tissue, a potential risk factor for carotid atherosclerosis: results of the Multicultural Community Health Assessment Trial (M-CHAT) Stroke 2007 38 9 2422 2429 10.1161/STROKEAHA.107.484113 17673711

[B12] Blackburn P Lemieux I Lamarche B Bergeron J Perron P Tremblay G Gaudet D Despres JP Hypertriglyceridemic waist: a simple clinical phenotype associated with coronary artery disease in women Metabolism 2012 61 56 64 10.1016/j.metabol.2011.05.017 21733531

[B13] Lemieux I Pascot A Couillard C Lamarche B Tchernof A Almeras N Bergeron J Gaudet D Tremblay G Prud’homme D Nadeau A Despres JP Hypertriglyceridemic waist: A marker of the atherogenic metabolic triad (hyperinsulinemia; hyperapolipoprotein B; small, dense LDL) in men? Circulation 2000 102 179 184 10.1161/01.CIR.102.2.179 10889128

[B14] Lemieux I Poirier P Bergeron J Almeras N Lamarche B Cantin B Dagenais GR Despres JP Hypertriglyceridemic waist: a useful screening phenotype in preventive cardiology? Can J Cardiol 2007 23 Suppl B 23B 31B 17932584 10.1016/s0828-282x(07)71007-3 PMC2794461

[B15] Sam S Haffner S Davidson MH D’Agostino RB Sr Feinstein S Kondos G Perez A Mazzone T Hypertriglyceridemic waist phenotype predicts increased visceral fat in subjects with type 2 diabetes Diabetes Care 2009 32 1916 1920 10.2337/dc09-0412 19592623 PMC2752928

[B16] Lei Z Dong Y Xu M Li J Wang X Wang N The hypertriglyceridemic-waist phenotype in relation to carotid artery atherosclerosis in patients with type 2 diabetes mellitus Chin J Endocrinol Metab 2012 28 1 21 23

[B17] Zhe X Bai Y Cheng Y Xiao H Wang D Wu Y Huang X Tian X Wang T Hypertriglyceridemic waist is associated with increased carotid atherosclerosis in chronic kidney disease patients Nephron 2012 122 146 152 23736857 10.1159/000351042

[B18] Bernal E Marin I Munoz A Saban J Sarabia F Garcia-Medina A Vicente T Cano A Hypertriglyceridemic waist phenotype is a risk factor for subclinical atherosclerosis in human immunodeficiency virus-infected patients Med Clin 2012 139 13 561 565 10.1016/j.medcli.2012.03.037 22985869

[B19] Lear SA Birmingham CL Chockalingam A Humphries KH Study design of the Multicultural Community Health Assessment Trial (M-CHAT): a comparison of body fat distribution in four distinct populations Ethn Dis 2006 16 96 100 16599355

[B20] Kriska AM Knowler WC LaPorte RE Drash AL Wing RR Blair SN Bennett PH Kuller LH Development of questionnaire to examine relationship of physical activity and diabetes in Pima Indians Diabetes Care 1990 13 401 411 10.2337/diacare.13.4.401 2318100

[B21] Pereira MA Kriska AM Joswiak ML Dowse GK Collins VR Zimmet PZ Gareeboo H Chitson P Hemrai F Purran A Physical inactivity and glucose intolerance in the multiethnic island of Mauritius Med Sci Sports Exerc 1995 27 1626 1634 8614318

[B22] Arsenault BJ Lemieux I Despres JP Wareham NJ Kastelein JJ Khaw KT Boekholdt SM The hypertriglyceridemic-waist phenotype and the risk of coronary artery disease: results from the EPIC-Norfolk prospective population study CMAJ 2010 182 1427 1432 10.1503/cmaj.091276 20643837 PMC2942915

[B23] Aminbakhsh A Frohlich J Mancini GBJ Detection of early atherosclerosis with B mode carotid ultrasonography: assessment of a new quantitative approach Clin Invest Med 1999 22 265 274 10664868

[B24] Blackburn P Lemieux I Almeras N Bergeron J Cote M Tremblay A Lamarche B Despres JP The hypertriglyceridemic waist phenotype versus the national cholesterol education program-adult treatment panel III and international diabetes federation clinical criteria to identify high-risk men with an altered cardiometabolic risk profile Metabolism 2009 58 8 1123 1130 10.1016/j.metabol.2009.03.012 19481769

[B25] Gazi IF Filippatos TD Tsimihodimos V Saougos VG Liberopoulos EN Mikhailidis DP Tselepis AD Elisaf M The hypertriglyceridemic waist phenotype is a predictor of elevated levels of small, dense LDL cholesterol Lipids 2006 41 7 657 654 10.1007/s11745-006-5015-8 17069348

[B26] Solati M Ghanbarian A Rahmani M Sarbazi N Allahverdian S Azizi F Cardiovascular risk factors in males with hypertriglyceridemic waist (Tehran Lipid and Glucose Study) Int J Obes Relat Metab Dis 2004 28 5 706 709 10.1038/sj.ijo.0802582 14770189

[B27] LaMonte MJ Ainsworth BE DuBose KD Grandiean PW Davis PG Yanowitz FG Durstine JL The hypertriglyceridemic waist phenotype among women Atherosclerosis 2003 171 1 123 130 10.1016/j.atherosclerosis.2003.07.008 14642414

[B28] Kahn HS Valdez R Metabolic risks identified by the combination of enlarged waist and elevated triacylglycerol concentration Am J Clin Nutr 2003 78 928 934 14594778 10.1093/ajcn/78.5.928

[B29] Tanko LB Bagger YZ Qin G Alexanders P Larsen PJ Christiansen C Enlarged waist combined with elevated triglycerides is a strong predictor of accelerated atherogenesis and related cardiovascular mortality in postmenopausal women Circulation 2005 111 15 1883 1890 10.1161/01.CIR.0000161801.65408.8D 15837940

[B30] Czernichow S Bruckert E Bertrais S Galan P Hercberg S Oppert JM Hypertriglyceridemic wasit and 7.5-year prospective risk of cardiovascular disease in asymptomatic middle-aged men Int J Obes 2007 31 5 791 796 10.1038/sj.ijo.0803477 17047639

[B31] St-Pierre J Lemieux I Perron P Brisson D Santure M Vohl MC Despres JP Gaudet D Relation of the "hypertriglyceridemic waist" phenotype to earlier manifestations of coronary artery disease in patients with glucose intolerance and type 2 diabetes mellitus Am J Cardiol 2007 99 3 369 373 10.1016/j.amjcard.2006.08.041 17261400

[B32] Zhang M Gao Y Chang H Wang X Liu D Zhu Z Huang G Hypertriglyceridemic-waist phenotype predicts diabetes: a cohort study in Chinese urban adults BMC Public Health 2012 12 1081 10.1186/1471-2458-12-1081 23241342 PMC3552698

[B33] Carlsson AC Riserus U Arnlov J Hypertriglyceridemic waist phenotype is associated with decreased insulin sensitivity and incident diabets in elderly men Obesity 2013 10.1002/oby.20434 10.1002/oby.20434 23512911

[B34] Despres JP Lemieux I Abdominal obesity and metabolic syndrome Nature 2006 444 881 887 10.1038/nature05488 17167477

[B35] Reaven G Insulin resistance and coronary heart disease in nondiabetic individuals ATVB 2012 32 1754 1759 10.1161/ATVBAHA.111.241885 22815340

[B36] St-Pierre J Lemieux I Vohl MC Perron P Tremblay G Despres JP Gaudet D Contribution of abdominal obesity and hypertriglyceridemia to impaired fasting glucose and coronary artery disease Am J Cardiol 2002 90 15 18 12088772 10.1016/s0002-9149(02)02378-0

[B37] Freedman DS Jacobsen SJ Barboriak JJ Sobocinski KA Anderson AJ Kissebah AH Sasse EA Gruchow HW Body fat distribution and male/female differences in lipids and lipoproteins Circulation 1990 81 1498 1506 10.1161/01.CIR.81.5.1498 2110035

[B38] Larsson B Bengtsson C Bjorntorp P Lapidus L Sjostrom L Syardsudd K Tibblin G Wedel H Welin L Wilhelmsen L Is abdominal body fat distribution a major explanation for the sex difference in the incidence of myocardial infarction? The study of men born in 1913 and the study ow women, Goteborg, Sweden Am J Epidemiology 1992 135 3 266 273 10.1093/oxfordjournals.aje.a116280 1546702

[B39] Gupta M Singh N Tsiqoulis M Kaiij M Hirjikaka S Quan A Teoh H Verma S Perceptions of Canadian primary care physicians towards cardiovascular risk assessment and lipid management Can J Cardiology 2012 28 1 14 19 10.1016/j.cjca.2011.09.014 22264843

[B40] Wang J Waist circumference: a simple, inexpensive, and reliable tool that should be included as part of physical examinations in the doctor’s office Am J Clin Nutr 2003 78 902 903 14594773 10.1093/ajcn/78.5.902

[B41] Yamada S Tsukamoto Y Irie J Waist circumference in metabolic syndrome Lancet 2007 370 9598 1541 1542 17980731 10.1016/S0140-6736(07)61656-0

[B42] Mason C Katzmarzyk PT Effect of the site measurement of waist circumference on the prevalence of the metabolic syndrome Am J Cardiol 2009 103 12 1716 1720 10.1016/j.amjcard.2009.02.018 19539081

[B43] Lin CC Yu SC Wu BJ Chang DJ Measurement of waist circumference at different sites affects the detection of abdominal obesity and metabolic syndrome among psychiatric patients Psychiatry Res 2012 197 3 322 326 10.1016/j.psychres.2011.09.012 22370155

[B44] Wang J Thornton JC Bari S Williamson B Gallagher D Heymsfield SB Horlick M Kotler D Laferrere B Mayer L Pi-Sunver FX Pierson RN Jr Comparisons of waist circumferences measured at 4 sites Am J Clin Nutr 2003 77 2 379 384 12540397 10.1093/ajcn/77.2.379

[B45] Lear SA Humphries KH Kohli S Chockalingam A Frohlich JJ Birmingham CL Visceral adipose tissue accumulation differs according to ethnic background: results of the Multicultural Community Health Assessment Trial (M-CHAT) Am J Clin Nutr 2007 86 2 353 359 17684205 10.1093/ajcn/86.2.353

[B46] Kohli S Sniderman AD Tchernof A Lear SA Ethnic-specific differences in abdominal subcutanepus tissue compartments Obesity 2010 18 11 2177 2183 10.1038/oby.2010.94 20448537

[B47] Kurian AK Cardarelli KM Racial and ethnic differences in cardiovascular disease risk factors: a systematic review Ethn Dis 2007 17 1 143 152 17274224

[B48] Chiu M Austin PC Manuel DG Tu JV Comparison of cardiovascular risk profiles among ethnic groups using population health surveys between 1996 and 2007 CMAJ 2010 182 8 E301 E310 10.1503/cmaj.091676 20403888 PMC2871219

[B49] Anand SS Yusuf S Vuksan V Devanesen S Teo KK Montague PA Kelemen L Yi C Lonn E Gerstein H Hegele RA McQueen M for the SHARE Investigators Differences in risk factors, atherosclerosis, and cardiovascular disease between ethnic groups in Canada: the Study of Health Assessment and Risk in Ethnic groups (SHARE) Lancet 2000 356 279 284 10.1016/S0140-6736(00)02502-2 11071182

