# Supplementary material for: The association between "hypertriglyceridemic waist" and sub-clinical atherosclerosis in a multiethnic population: a cross-sectional study
Source: Lipids Health Dis. 2014 Feb 23;13:38. doi: 10.1186/1476-511X-13-38 (PMC3938067; doi:10.1186/1476-511X-13-38)
Supplement: Additional file 1 — The association of HTGW with IMT, total area and presence of carotid artery plaques in men. [file 1476-511X-13-38-S1.doc]

Additional file 1. The association of HTGW with IMT, total area and presence of carotid artery plaques in men

| **A** | IMT |  | Total area |  | Plaques |  |
| --- | --- | --- | --- | --- | --- | --- |
|  | B (95%CI)* | *p* value | B (95%CI)* | *p* value | OR (95%CI) | *p* value |
| Elevated TG vs. Without HTGW | -0.025  (-0.087, 0.042) | 0.453 | 0.106  (-0.085, 0.336) | 0.297 | 1.547  (0.679, 3.523) | 0.299 |
| Elevated WC vs. Without HTGW | 0.059  (0.017, 0.102) | 0.005 | 0.124  (0.001, 0.261) | 0.047 | 1.260  (0.739, 2.148) | 0.395 |
| HTGW vs. Without HTGW | 0.084  (0.037, 1.133) | < 0.001 | 0.202  (0.058, 0.366) | 0.005 | 1.904  (1.040, 3.486) | 0.037 |

| **B** | IMT |  | Total area |  | Plaques | men |
| --- | --- | --- | --- | --- | --- | --- |
|  | B (95%CI) | *p* value | B (95%CI) | *p* value | OR (95%CI) | *p* value |
| Elevated TG vs. Without HTGW | -0.048  (-0.113, 0.019) | 0.159 | 0.043  (-0.144, 0.270) | 0.678 | 1.376  (0.563, 3.365) | 0.484 |
| Elevated WC vs. Without HTGW | 0.029  (-0.021, 0.082) | 0.252 | 0.148  (-0.005, 0.324) | 0.059 | 1.327  (0.676, 2.606) | 0.411 |
| HTGW vs. Without HTGW | 0.009  (-0.051, 0.073) | 0.765 | 0.093  (-0.081, 0.302) | 0.314 | 1.628  (0.714, 3.713) | 0.247 |

Elevated waist circumference (WC) was ≥ 90cm and elevated triglyceride (TG) levels were ≥ 2mmol/L. Outcomes variables: IMT (intima-media thickness), total area (a measure of total atherosclerotic burden), and presence of plaques. A: Model 1: adjusted for age, ethnicity, smoking, and physical activity. B: Model 2 = Model 1 + additional adjustment for systolic blood pressure, total cholesterol, HDL-C, fasting blood glucose, family history of CVD, and BMI. * presented as b^e^ – 1 and interpreted (after multiplied by 100) as percent change in the outcome (IMT, total area) for each unit change in the independent variable.
