# Supplementary material for: The association between "hypertriglyceridemic waist" and sub-clinical atherosclerosis in a multiethnic population: a cross-sectional study
Source: Lipids Health Dis. 2014 Feb 23;13:38. doi: 10.1186/1476-511X-13-38 (PMC3938067; doi:10.1186/1476-511X-13-38)
Supplement: Additional file 2 — The association of HTGW with IMT, total area and presence of carotid artery plaques in women. [file 1476-511X-13-38-S2.doc]

Additional file 2. The association of HTGW with IMT, total area and presence of carotid artery plaques in women

| **A** | IMT |  | Total area |  | Plaques |  |
| --- | --- | --- | --- | --- | --- | --- |
|  | B (95%CI) | *p* value | B (95%CI) | *p* value | OR (95%CI) | *p* value |
| Elevated TG vs. Without HTGW | 0.051  (0.013, 0.090) | 0.007 | 0.076  (-0.037, 0.200) | 0.192 | 0.938  (0.492, 1.790) | 0.847 |
| Elevated WC Without HTGW | 0.045  (0.015, 0.077) | 0.003 | 0.049  (-0.039, 0.145) | 0.287 | 0.935  (0.554, 1.577) | 0.800 |
| HTGW vs. Without HTGW | 0.041  (0.006, 0.077) | 0.020 | 0.115  (0.006, 0.235) | 0.037 | 1.435  (0.778, 2.647) | 0.248 |

| **B** | IMT |  | Total area |  | Plaques | women |
| --- | --- | --- | --- | --- | --- | --- |
|  | B (95%CI) | *p* value | B (95%CI) | *p* value | OR (95%CI) | *p* value |
| Elevated TG vs. Without HTGW | 0.029  (-0.010, 0.069) | 0.143 | 0.006  (-0.104, 0.129) | 0.925 | 0.657  (0.323, 1.339) | 0.248 |
| Elevated WC vs. Without HTGW | 0.016  (-0.021, 0.054) | 0.388 | 0.035  (-0.073, 0.156) | 0.542 | 0.809  (0.417, 1.568) | 0.530 |
| HTGW vs. Without HTGW | -0.001  (-0.044, 0.045) | 0.980 | 0.053  (-0.077, 0.203) | 0.440 | 0.992  (0.447, 2.201) | 0.984 |

Elevated waist circumference (WC) was ≥ 85cm and elevated triglyceride (TG) levels were ≥ 1.5mmol/L. Outcomes variables: IMT (intima-media thickness), total area (a measure of total atherosclerotic burden), and presence of plaques. Table A: Model 1: adjusted for age, ethnicity, smoking, and physical activity. B: Model 2: Model 1 + additional adjustment for systolic blood pressure, total cholesterol, HDL-C, fasting blood glucose, family history of CVD, and BMI. * presented as b^e^ – 1 and interpreted (after multiplied by 100) as percent change in the outcome (IMT, total area) for each unit change in the independent variable.
